# Cajachalcone: An Antimalarial Compound from *Cajanus cajan* Leaf Extract

**DOI:** 10.1155/2013/703781

**Published:** 2013-07-18

**Authors:** E. O. Ajaiyeoba, O. O. Ogbole, O. O. Abiodun, J. S. Ashidi, P. J. Houghton, C. W. Wright

**Affiliations:** ^1^Department of Pharmacognosy, University of Ibadan, Ibadan 200284, Nigeria; ^2^Department of Pharmacology & Therapeutics, University of Ibadan, Ibadan 200284, Nigeria; ^3^Department of Biological Sciences, Olabisi Onabanjo University, Ago-Iwoye 110001, Nigeria; ^4^Department of Pharmacy, King's College London, 150 Stamford Street, London SE1 8WA, UK; ^5^The School of Pharmacy, University of Bradford, West Yorkshire BD7 1DP, UK

## Abstract

*Cajanus cajan* L, a member of the family Fabaceae, was identified from the Nigerian antimalarial ethnobotany as possessing antimalarial properties. The bioassay-guided fractionation of the crude methanol extract of *C. cajan* leaves was done *in vitro* using the multiresistant strain of *Plasmodium falciparum* (K1) in the parasite lactate dehydrogenase assay. Isolation of compound was achieved by a combination of chromatographic techniques, while the structure of the compound was elucidated by spectroscopy. This led to the identification of a cajachalcone, 2′,6′-dihydroxy-4-methoxy chalcone, as the biologically active constituent from the ethyl acetate fraction. Cajachalcone had an IC_50_ value of 2.0 **μ**g/mL (7.4 **μ**M) and could be a lead for anti-malarial drug discovery.

## 1. Introduction

Malaria is a vector borne disease, caused by the Plasmodium parasite. According to WHO report, there were estimated 216 million episodes of malaria in 2010, of which approximately 81%, or 174 million cases, were in the African region. There were estimated 655,000 malaria deaths in 2010, of which 91% were in Africa. Approximately 86% of malaria deaths globally were of children under 5 years of age [1]. In addition to acute disease episodes and deaths in Africa, malaria also contributes significantly to anaemia in children and pregnant women, adverse birth outcomes such as spontaneous abortion, stillbirth, premature delivery, and low birth weight, and overall child mortality. 

Included in the WHO report was the fact that resistance to artemisinin, a vital component of drugs used in the treatment of *P. falciparum *malaria, has been reported in a growing number of countries in Southeast Asia. Resistance to pyrethroids, the insecticides used in ITNs and most commonly used in IRS, has been reported in 27 countries in Africa and 41 countries worldwide [1]. Unless properly managed, such resistance potentially threatens future progress in malaria control. The search for new antimalarial drugs requires identification of new biochemical targets for drug development and development of new chemical entities [2, 3]. 

Epidemiological studies have provided convincing evidence that natural dietary compounds, which humans consume as food, possess many biological activities [4]. One plant food that has been shown to be therapeutic against a number of diseases is pigeon pea, *Cajanus cajan* L. (Fabaceae), an important grain legume crop in the tropics and subtropics. The extracts of pigeon pea are commonly used to treat diabetes, fever, dysentery, hepatitis, and measles worldwide [5, 6]. *Cajanus cajan* has been used traditionally as a laxative and was identified as an antimalarial remedy [7]. In continuation of our study of the Nigerian ethnomedicine for the discovery of new antimalarial drugs [7, 8], the present report is on the bioassay-guided fractionation and isolation of antiplasmodial compounds from *Cajanus cajan* leaf extract.

## 2. Materials and Method

### 2.1. Plant Collection and Authentication

*Cajanus cajan *leaves were collected from Otu, Oyo State of Nigeria, in the month of January and authenticated at the Herbarium of Botany Department, University of Ibadan (UI), and that of the Forestry Research Institute of Nigeria (FRIN), Ibadan, where a voucher specimen was deposited as FHI 106560.

### 2.2. Plant Extraction & Fractionation

Leaves of *C. cajan* were air dried at RT (26–31°C) and pulverized with a hammer mill. 500 g of plant material was extracted in redistilled methanol (2.0 L) by maceration at RT (30°C) for 72 h. After determination of yield of crude methanol extract, the sample was stored in the fridge (4°C) till needed for analysis.

### 2.3. Isolation of Compounds

2.0 g of dry weight of crude methanol extract was fractionated by suspension in MeOH : H_2_O in a ratio of 70 : 30 to yield 0.35 g of hexane, 0.46 g of dichloromethane (DCM), 0.41 g of EtOAc, and 0.73 g of aqueous methanol fractions, respectively. The hexane and DCM fractions were combined based on the analysis and chromatographed on flash column using silica gel (Merck). It was eluted with increasing polarity of hexane-DCM, and 50 mL portions were collected, respectively. The fractions that eluted with hexane : DCM (50 : 50 to 20 : 80; 130 mg) indicated the presence of predominantly 3 compounds on TLC analysis. This was subjected to PTLC using CHCl_3_ : EtOAc (17 : 3) (Merck, 20 × 20 cm, 12 plates) to give compounds 1, 2, and 3 with *R*_*f*_ 0.45, 0.55, and 0.80, respectively. The compounds were subjected to structural analysis using NMR and MS.

### 2.4. Antiplasmodial Assay

The asexual stages of *Plasmodium falciparum* (multidrug resistant strain K1) obtained from Dr. Warhurst, London School of Hygiene and Tropical Medicine, were cultured continuously according to the modified candle jar method [9]. The method of Makler and Hinrichs [10] was used in the estimation of parasite growth inhibition. Cultures were cryopreserved to contain at least 5% ring-form parasites and were maintained at 2–4% hematocrit; this was used in preparing 2% hematocrit and washing with phosphate buffered solution (PBS) 3 times. Stock solutions of extracts were prepared by dissolving known quantities of dried extracts (500 *μ*g) in 1 : 1 dimethyl sulphoxide (250 *μ*L) and distilled water (250 *μ*L). Serial dilutions (10 dilutions, 0.5–500 *μ*g/mL) of the extracts/fractions were made in quadruplicates in 96-well microtitre plates.

The drug plate was placed in the chamber with a little sterile water in a Petri dish. This was placed in the laminar flow chamber (Envair, UK) gassed with prefiltered mixture of 3% O_2_, 4% CO_2_, and 93% N_2_, and then swiftly sealed and incubated at 37°C for 48 hours. After incubation, acetylpyridine adenine dinucleotide (APAD) regent was added to each well, followed by N-bromosuccinimide (NBS) and then incubated at 37°C for 20 min [10]. Optical density was measured in a plate reader at 550 nm and analysed with a Wallac counter using an MS excel program. IC_50_ values were estimated by plotting the % inhibition against the log drug concentration at 95% confidence limits using the linear and nonlinear regression analyses. 

## 3. Results

The crude methanol extract (dry weight yield of 8.6 g) had an IC_50_ of 53.5 *μ*g/mL, the hexane fraction had IC_50_ of 62.5, and both DCM and aqueous MeOH had IC_50_ of 31.3 *μ*g/mL, while Ethyl Acetate fraction had IC_50_ of 15.6 *μ*g/mL compared to chloroquine diPO4 with IC_50_ 0.21 *μ*g/mL (0.66 *μ*M).

Yields of compounds 1–3 were 5.0 mg (3.8%), 7.0 mg (5.3%), and 11.3 mg (8.7%) with IC_50_ values of 2.0 *μ*g/mL (7.40 *μ*M), 5.4 *μ*g/mL, and 5.6 *μ*g/mL, respectively (see Table 1 for details).

**Figure 1:**
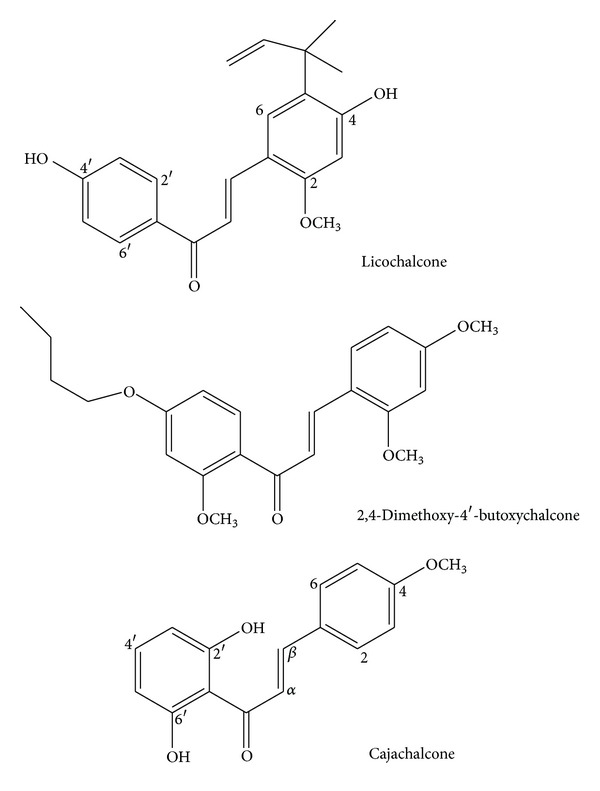
Structures of chalcones.

Compound 1 obtained from chromatographic analysis of the ethyl acetate fraction had an IC_50_ of 2.0 *μ*g/mL. The EI-MS of compound 1 had the [M+] at m/z 270, and C-13 NMR broad band indicated the presence of 16 carbon atoms and in agreement with C_16_H_14_O_4_. Comparison of the spectroscopic data with those obtained from the literature identified the compound as 2′,6′-dihydroxy-4-methoxy chalcone (cajachalcone) (Figure 1). Compound 2 with an IC_50_ > 5 *μ*g/mL also obtained from the ethyl acetate fraction, its EI-MS had a molar mass of 294, the C-13 NMR, indicated 19C atoms and a formula of C_19_H_17_O_3_, suggestive of a phenanthrone furandione derivative. The data available were not sufficient to confirm the structure of compounds 2 and 3. 

## 4. Discussion

*Cajanus cajan* L., Fabaceae, has been used locally as part of ethnotherapy for malaria infection in south western Nigeria; its utilization as an antimalarial agent cuts across the whole of Sub-Saharan Africa as well as other tropical countries as reported by some authors [7, 11]. From the result of this study, the crude methanol extract of this plant had an IC_50_ of 53.5 *μ*g/mL; subsequently, bioassay-guided fractionation and chromatographic separations led to the isolation of the compound responsible for the displayed antimalarial activity 2′,6′-dihydroxy-4-methoxy chalcone (cajachalcone); the compound displayed significant antimalarial activity IC_50_ of 2.0 *μ*g/mL (7.4 *μ*M). Chloroquine diphosphate (10 *μ*g/mL) was used as control and had IC_50_ value of 0.21 *μ*g/mL (0.66 *μ*M). Its structure was confirmed by comparison with ^1^H-NMR data reported for licochalcone A and 2,4-dimethoxy-4-butoxychalcone as shown in Table 2 [12, 13].

Naturally occurring chalcones (1,3-diaryl-2-propen-1-one) are the key intermediates for various plant metabolites. They are biologically active compounds with known antibacterial [14, 15], antifilarial [16], antiviral [17, 18], antileishmanial [19], and cytotoxic [20, 21] activities.

Chalcone synthesis by shikimate pathway is straightforward. Licochalcone A, an oxygenated chalcone (Figure 1) first isolated from roots of Chinese licorice, showed antimalarial activity in both *in vitro* and *in vivo* systems [22]. Since then, investigators have been searching for new more-potent lead molecules based on chalcone scaffolds as potential antimalarial agents [23, 24]. 

The simple structure and unambiguous synthesis of chalcones have attracted the attention of chemists to develop different analogs of this novel scaffold for various infectious diseases including malaria. A series of alkoxylated, hydroxylated, prenylated, oxygenated, quinolylated chalcones from natural sources and syntheses have been evaluated for antiplasmodial activity with encouraging results [25, 26].

Using Claisen-Schmidt condensation method, Yadav et al. [27] synthesized 4-methoxy; 2,4-dimethoxy; 2,5-dimethoxy; 3,4-dimethoxy and 3,4,5-trimethoxy benzaldehyde series of chalcone derivatives. In the 4-methoxy series, with IC_50_ of 1.6 *μ*g/mL, the antimalarial activity compared favourably with licochalcone A (IC_50_ of 1.43 *μ*g/mL) against chloroquine-sensitive 3D7 strain [22]. In 2,4-dimethoxy series, IC_50_ values of between 1.1 and 7.68 *μ*g/mL were obtained. 

The antimalarial activity of 2,4-dimethoxy chalcone IC_50_ 2.1 *μ*g/mL (a naturally occurring 4-methoxy derivative) in our study was also compared favourably with the result of synthesized 4-methoxy series (IC_50_ 1.6 *μ*g/mL). Meanwhile, Yadav and coworkers [27] concluded that the presence of methoxy groups at positions 2 and 4 in chalcone derivatives (Figure 1) appeared to be favorable for antimalarial activity as compared to other methoxy-substituted chalcones; thus, we can infer that the isolated chalcone could be a template for the synthesis of 2,4-dimethoxy substituted derivatives, with methoxy substitution at position C-4. 

It is believed that chalcone derivatives that possess antimalarial activity interact with parasite *P. falciparum* enzyme cysteine protease, one of the key enzymes involved in hemoglobin degradation within the acidic food vacuole of the intraerythrocytic parasite [28]. Inhibition of this enzyme hampers digestion of hemoglobin within the food vacuole and proves fatal for the parasite. 

The World Health Organization 2011 [1] has advised that the development of new tools is a necessary priority, particularly for vector control, diagnostic testing, treatment, and surveillance. It is our belief that 2,4-dimethoxy chalcone isolated from *Cajanus cajan* L could be a lead for antimalarial drug development.

## 5. Conclusion

*Cajanus cajan *is a common food and medicinal plant in the tropical Africa. Its leaf extract has furnished a chalcone, as the antimalarial component. Chalcones and derivatives are small bioactive molecules that have been synthesized and so have a high potential as leads for discovery and development of antimalarial agents. 

## Figures and Tables

**Table 1:** Yield and *in vitro* antiplasmodial activity of *Cajanus cajan* leaf fractions and compounds.

Fractions/compounds/drug	Yield (%)	IC_50_ values with *P. falciparum*, K1 in *µ*g/mL (*µ*M)
Hexane	17.5	62.5
Dichloromethane	23.0	31.3
Ethyl acetate	20.5	15.6
Aq. methanol	36.5	31.3
Compound 1 (chalcone)	3.8	2.0 (7.40)
Compound 2	5.3	5.4 (18.37)
Compound 3	8.7	5.6
Chloroquine phosphate		0.2 (0.66)

**Table 2:** Proton-^1^H NMR data of chalcones^a^.

Position	Licochalcone^b^	Butoxychalcone^c^	Cajachalcone
2	6.43 (1H, s)	3.85 (3H, s, OMe)	6.90 (2H, d, *J = *8.5, H2, H6)

3		6.47 (1H, d, *J = *2.3)	7.35 (2H, d, *J = * 8.5, H3, H5)
	6.19 (1H, dd, *J = *10, 18)		
	5.31 (1H, d, *J = *10, H_B_)		
	5.34 (1H, d, *J = *18, H_C_)		

4		3.86 (3H, s, OMe)	3.80 (3H, s, OMe)

5	7.45 (1H, s)		

6	3.81 (3H, s, OMe)		

*α*	7.53 (1H, d, *J* = 15)	7.56 (1H, d, *J = *15.7)	7.54 (1H, d, *J = *15.3)

*β*	8.03 (1H, d, *J* = 15)	8.04 (1H, d, *J = *15.7)	8.05 (1H, d, *J = *15.3)

1′			

2′, 6′	7.97 (2H, d, *J* = 8.5)	8.02 (2H, m)	

3′, 5′	6.97 (2H, d, *J* = 8.5)	6.95 (2H, m)	8.45 (2H, d, *J = *8.7)

4′			6.40 (1H, dd, *J = *2.5, 2.5)
		4.04 (2H, t, *J = *6.4, H1′′)	
		1.78 (2H, m, H2′′)	
		0.99 (3H, t, *J = *7.4, H4′′)	

^a^ *J* values are in Hertz.

^b^Saitoh and Shibata, 1975 [12].

^c^Chen et al., 1997 [13].
